# Field-Testing the Euro-MCD Instrument: Important Outcomes According to Participants Before and After Moral Case Deliberation

**DOI:** 10.1007/s10730-020-09421-9

**Published:** 2020-08-08

**Authors:** J. C. de Snoo-Trimp, A. C. Molewijk, M. Svantesson, G. A. M. Widdershoven, H. C. W. de Vet

**Affiliations:** 1 Amsterdam UMC, Vrije Universiteit Amsterdam, Department of Ethics, Law and Humanities, Amsterdam Public Health Research Institute, De Boelelaan 1117, Amsterdam, The Netherlands; 2 Center for Medical Ethics, University of Oslo, Oslo, Norway; 3 Faculty of Medicine and Health, University Health Care Research Center, Örebro University, Örebro, Sweden; 4 Amsterdam UMC, Vrije Universiteit Amsterdam, Department of Epidemiology and Biostatistics, Amsterdam Public Health Research Institute, De Boelelaan 1117, Amsterdam, The Netherlands

**Keywords:** Clinical ethics support, Moral case deliberation, Evaluation, Outcomes, Factor analyses

## Abstract

Ethics support services like Moral Case Deliberation (MCD) intend to support healthcare professionals in ethically difficult situations. To assess outcomes of MCD, the Euro-MCD Instrument has been developed. Field studies to test this instrument are needed and have been conducted, examining important outcomes before MCD participation and experienced outcomes. The current study aimed to (1) describe how participants’ perceive the importance of MCD outcomes after MCD; (2) compare these perceptions with those before MCD participation; and (3) test the factor structure of these outcomes. Swedish, Norwegian and Dutch healthcare professionals rated the importance of outcomes in the Euro-MCD Instrument after four and eight MCDs. Ratings were compared with those before MCD participation using paired and independent samples t-tests. The factor structure was tested using exploratory factor analyses. After 4 and 8 MCDs, 443 respectively 247 respondents completed the instrument. More than 69% rated all MCD outcomes as ‘quite’ or ‘very’ important, especially outcomes from Enhanced Collaboration, Improved Moral Reflexivity and Improved Moral Attitude. Significant differences for 16 outcomes regarding ratings before and after MCD participation were not considered meaningful. Factor analyses suggested three categories, which seemingly resemble the domains Improved Moral Reflexivity, Enhanced Collaboration and a combination of Improved Moral Attitude and Enhanced Emotional Support. After participation in MCDs, respondents confirmed the importance of outcomes in the Euro-MCD Instrument. The question on perceived importance and the categorization of outcomes need reconsideration. The revised instrument will be presented elsewhere, based on all field studies and theoretical reflections.

## Introduction

In the past decades, ethics support services have rapidly been developed in many healthcare settings and institutions (Molewijk et al. 2017). These services aim to support healthcare professionals in dealing with ethical dilemmas and situations in which they are uncertain or disagree about what good care would entail. In several European healthcare settings, this support is provided in the form of Moral Case Deliberation (MCD). In an MCD, participants jointly elaborate on an ethically difficult situation under guidance of a facilitator (Molewijk et al. 2008). After formulating a moral question about the situation, the group explores relevant facts, norms, values and alternatives in order to come at a decision, common ground, or new insights into the situation (Molewijk et al. 2008).

The increasing implementation of MCD gives reason to study what outcomes MCD leads to. Does it—according to its goals—indeed support healthcare professionals in dealing with ethically difficult situations and in what way? Insights into how healthcare professionals—the actual end-users—benefit (or not) from participation in MCD is needed to further improve the MCD as a supportive service for them and to show its value and quality to healthcare organizations that want to implement it (Schildmann et al. 2019; Wäscher et al. 2017; Craig and May 2006). As stated by Craig and May (2006, p. 168), there is a need for evaluation research notwithstanding the inherent and theoretical benefit of clinical ethics support (CES): “As bioethicists, we are well aware of the theoretical goods such [CES] services might achieve, but should insist on evidence regarding the effectiveness of ethics consultation relative to these goods.”

Several evaluation studies showed—in general—positive results (De Snoo-Trimp et al. 2019; Haan et al. 2018; Spijkerboer et al. 2017; Bartholdson et al. 2017; Seekles et al. 2016; Silén et al. 2015; Weidema et al. 2013, 2015; Lillemoen and Pedersen 2015; Janssens et al. 2014; Hem et al. 2014). These studies all focused on the satisfaction of healthcare professionals regarding the sessions themselves as well as their experiences beyond MCD in daily practice, with use of self-reported questionnaires, interviews and focus groups (Haan et al. 2018). For instance, in the study by Bartholdson et al. (2017), participants of ethics case reflection sessions (similar to MCD) were interviewed about enablers and barriers for clarifying perspectives, based on their experiences from attending the sessions. In another study (Weidema et al. 2013), healthcare professionals completed an evaluation questionnaire after each MCD session in which they had to rate the quality of the session and related elements of the session like atmosphere and relevance of the moral issue. In the review by Haan et al. (2018), empirical evidence for impact of MCD was systematically studied. They concluded that in the included studies “most reported changes were considered positive.” Notwithstanding the positive findings, evaluation research in MCD and other types of clinical ethics support is still an underdeveloped area, as only few systematic comparable research studies have been done and only few structural evaluation tools exist (Schildmann et al. 2019; Haan et al. 2018). Schildmann et al. (2019) recently described that, despite the increasing attention for quality of CES services, “there has been a paucity of evidence on the outcomes of CES [services], and considerable controversy regarding the contribution of CES [services] to clinical practice.” Hence, there is a need for thorough and systematic research on methods for MCD evaluation.

In this evaluation research, it is important to give attention to the perspectives of participants. In the end, they are the users of this CES service. It would make no sense, for instance, to evaluate such a service only based on what clinical ethicists or managers would consider important outcomes, because it might well be that a CES service leads to these outcomes while healthcare professionals might still not feel supported in their daily morally-challenging practice. Information about what outcomes participants define as important could further be used to tailor the implementation and the content of the CES service to participants’ needs and expectations. Craig and May (2006) already warned of the danger of evaluating CES with inappropriate criteria like objective and predetermined standards or solely satisfaction rates. A bottom-up approach to evaluation involving active involvement of relevant stakeholders has been recommended (Wäscher et al. 2017; Schildmann et al. 2012). Therefore, we are interested in input from MCD participants working in healthcare practice here: how do they think about the importance of (possible) outcomes of MCD? As a response to the needs for systematic CES evaluation research, and the lack of focus on participants’ perspectives on outcomes in the field of MCD, the Euro-MCD Instrument was developed (Svantesson et al. 2014).

### The Euro-MCD Instrument

The Euro-MCD Instrument aims to measure outcomes of MCD by presenting 26 possible outcomes and assessing *perceptions of importance* and self-reported *experiences* of these outcomes during the sessions and in daily practice according to participants (Svantesson et al. 2014; see also Tables 1, 2)*.* The instrument is a questionnaire containing 26 possible outcomes of MCD and asks for each one to rate the perceived importance and/or experience. The rating for perceived importance (‘How important is the outcome to you?’) concerns a 1–4 point Likert scale: 1 ‘Not important’; 2 ‘Somewhat important’; 3 ‘Quite important’ and 4 ‘Very important’. The answer option ‘Cannot take stand’ can also be chosen. The results for the question on experience are published elsewhere (De Snoo-Trimp et al. 2019). It further contains an open question asking for possible important outcomes according to the respondents and a question to rank the five most important outcomes from the list. The instrument was developed in a comprehensive and systematic process including literature review, a Delphi Expert-Panel from various countries and content validity testing in the Netherlands, Norway and Sweden. The developers considered participants’ perceptions of importance as an essential step in further validating the instrument: “the specific context should have a say in which specific goals and outcomes of MCD are important” (Svantesson et al. 2014). For this further validation in field studies was said to be needed.

**Table 1:** Perceived importance of MCD outcomes before and after participation

No	Question: How important is the outcome to you? % of respondents per answer option	Not	Some what	Quite	Very	More or less important after MCD participation than before?^b^	
1	**Develops my skills to analyze ethical difficult situations**	**Before**	1	8	47	44	LESS*^
		**After**	2	16	47	35	
2	**More open communication among co-workers**	**Before**	0	4	34	62	LESS*^
		**After**	1	8	41	50	
3	**Consensus is gained amongst co-workers in how to manage the situation**	**Before**	2	10	46	42	LESS*^
		**After**^**a**^	2	17	46	35	
4	Enables me to better manage the stress from the ethical situation	**Before**	5	17	41	37	
		**After**^**a**^	4	20	44	32	
5	Contributes to the development of practice/policies in the workplace	**Before**	1	13	49	36	LESS^
		**After**^**a**^	2	18	47	33	
6	Gives me more courage to express my ethical standpoint	**Before**	4	21	47	28	
		**After**^**a**^	5	20	43	32	
7	I feel more secure to express doubts or uncertainty regarding difficult situations	**Before**	4	19	43	34	
		**After**^**a**^	4	18	44	34	
8	**Better mutual understanding of each other’s reasoning and acting**	**Before**	0	6	40	54	LESS*^
		**After**	1	11	43	45	
9	**I see the situation from different perspectives**	**Before**	0	9	44	47	LESS*^
		**After**^**a**^	1	15	47	36	
10	**I and my co-workers become more aware of recurring situations**	**Before**	1	10	47	42	LESS^
		**After**^**a**^	1	16	46	37	
11	Increases my awareness of the complexity of the situation	**Before**	2	17	46	35	
		**After**^**a**^	2	17	48	33	
12	Enhances my understanding of ethical theories	**Before**	3	21	46	30	LESS^
		**After**^**a**^	5	26	43	26	
13	Enables to decide on concrete actions to manage the situation	**Before**	1	6	44	48	LESS*^
		**After**^**a**^	2	16	48	34	
14	**Greater opportunity for everyone to have their say**	**Before**	3	16	43	38	
		**After**^**a**^	1	16	45	38	
15	**Enhances possibility to share difficult emotions and thoughts**	**Before**	0	13	44	43	
		**After**^**a**^	2	14	44	40	
16	**Find more courses of action to manage the situation**	**Before**	0	10	46	44	LESS*^
		**After**	2	13	51	34	
17	**I listen more seriously to other’s opinions**	**Before**	3	18	43	36	
		**After**^**a**^	2	14	42	42	
18	Increases awareness of own emotions	**Before**	3	19	44	34	
		**After**^**a**^	5	20	41	34	
19	Strengthens my self-confidence when managing difficult situations	**Before**	3	14	44	39	LESS*
		**After**^**a**^	4	19	45	32	
20	Develops my ability to identify the core ethical question in difficult situations	**Before**	1	12	48	39	LESS*^
		**After**^**a**^	3	19	47	31	
21	I and my co-workers examine more critically existing practice/policies in workplace	**Before**	2	14	50	34	LESS*^
		**After**^**a**^	3	23	46	28	
22	I and my co-workers manage disagreements more constructively	**Before**	1	12	46	41	LESS*^
		**After**^**a**^	2	17	45	36	
23	I gain more clarity about own responsibility in difficult situations	**Before**	1	13	47	39	LESS*^
		**After**^**a**^	2	20	44	34	
24	**Enhances mutual respect amongst co-workers**	**Before**	2	9	40	49	LESS*^
		**After**^**a**^	2	14	40	44	
25	I become more aware of my preconceived notions	**Before**	3	17	40	40	
		**After**^**a**^	4	18	42	36	
26	**I understand better what it means to be a good professional**	**Before**	4	16	42	39	

Bold items: top 10 of outcomes perceived by most respondents as quite or very important in either data prior to MCD participation, or after 4/8 sessions, or both

^a^More than 10% of respondents answered the option ‘Cannot take stand’ or did not give any answer; ^b^Only significant differences are shown: *significant in independent samples t-test (Chi Square), with respondents prior to MCD participation (N = 515) vs. respondents after MCD participation (N = 288), p-value < 0.05; ^^^significant in dependent samples t-test (Wilcoxon signed-rank test), with 273 respondents who rated items both before and after 4/8 MCD sessions, p-value < 0.05

**Table 2:** Correlations between items Euro-MCD Instrument

Item		Responses before or after MCD participation	Cluster		
			1	2	3
1	Develops my skills to analyze ethical difficult situations	Before		0.617	
		After		0.614	
2	More open communication among co-workers	Before			0.700
		After			0.662
3	Consensus is gained amongst co-workers in how to manage the situation	Before	–	–	–
		After			0.718
4	Enables me to better manage the stress from the ethical situation	Before	0.658		
		After	–	–	–
5	Contributes to the development of practice/policies in the workplace	Before		0.521	
		After			0.622
6	Gives me more courage to express my ethical standpoint	Before	0.738		
		After			0.515
7	I feel more secure to express doubts or uncertainty regarding difficult situations	Before	0.734		
		After			0.547
8	Better mutual understanding of each other’s reasoning and acting	Before			0.617
		After			0.667
9	I see the situation from different perspectives	Before		0.587	
		After		0.574	0.566
10	I and my co-workers become more aware of recurring situations	Before		0.588	
		After		0.585	0.550
11	Increases my awareness of the complexity of the situation	Before		0.550	
		After		0.719	
12	Enhances my understanding of ethical theories	Before		0.568	
		After		0.770	
13	Enables to decide on concrete actions to manage the situation	Before		0.565	
		After			0.592
14	Greater opportunity for everyone to have their say	Before			0.568
		After			0.559
15	Enhances possibility to share difficult emotions and thoughts	Before			0.574
		After	0.639		
16	Find more courses of action to manage the situation	Before		0.652	
		After		0.579	
17	I listen more seriously to other’s opinions	Before	0.571		0.509
		After	0.612		
18	Increases awareness of own emotions	Before	0.718		
		After	0.648	0.524	
19	Strengthens my self-confidence when managing difficult situations	Before	0.750		
		After	0.636	0.538	
20	Develops my ability to identify the core ethical question in difficult situations	Before		0.622	
		After		0.655	
21	I and my co-workers examine more critically existing practice/policies in workplace	Before		0.567	
		After	0.611		
22	I and my co-workers manage disagreements more constructively	Before			0.627
		After	0.684		
23	I gain more clarity about own responsibility in difficult situations	Before	–	–	–
		After	0.579	0.608	
24	Enhances mutual respect amongst co-workers	Before			0.722
		After	0.679		0.515
25	I become more aware of my preconceived notions	Before	0.549		
		After	0.586	0.518	
26	I understand better what it means to be a good professional	Before	0.626		
		After	0.681		

### Previous field studies with the Euro-MCD Instrument

Since 2014, several field studies have been conducted in Sweden, Norway and the Netherlands, using the Euro-MCD Instrument to assess what outcomes healthcare professionals perceive as important before participation in MCD (De Snoo-Trimp et al. 2017; Svantesson et al. 2019) and what outcomes they experience during the sessions and afterwards in daily practice (De Snoo-Trimp et al. 2019). In the latter study, factor analyses were performed to examine which outcomes highly correlate with each other and can be considered one domain.

However, the factor structure of MCD outcomes with regards to their perceived importance has not yet been examined. This is needed to gain additional insight in possible categorization of outcomes, because correlations among the various outcomes might be different when respondents rate importance of outcomes instead of whether (or not) they experienced the outcomes. In the Euro-MCD Instrument, the 26 possible outcomes were categorized into 6 domains: (1) Enhanced Emotional Support; (2) Enhanced Collaboration; (3) Improved Moral Reflexivity; (4) Improved Moral Attitude; (5) Impact on Organizational Level and (6) Concrete Results. This categorization was based on theoretical thinking by the Euro-MCD Research Team and the Delphi Panel (Svantesson et al. 2014). It is important to get empirical evidence about the structure of the data and explore meaningful dimensions. Furthermore, factor analysis informs about possible item reduction, i.e., deletion of outcomes, which do not correlate with any other outcomes (De Vet et al. 2011).

This study is part of the larger field study on validating and revising the Euro-MCD Instrument (Svantesson et al. 2014). To contribute to further validation of the Euro-MCD Instrument, the current study has three aims: (1) to examine how MCD participants perceive the importance of MCD outcomes *after* participating in MCD sessions; (2) to compare these perceptions with the perceived importance asked *before* participating in MCD sessions; and (3) to test the factor structure of these outcomes to further validate the instrument.

## Methods

### Design

This quantitative study had a descriptive and comparative design.

### Sampling and Data Collection

The Euro-MCD Instrument (Svantesson et al. 2014) was distributed among healthcare professionals in various healthcare settings in Sweden, Norway and the Netherlands. These healthcare professionals were recruited by convenience sampling of healthcare institutions that planned to organize a series of four to eight MCDs on a monthly basis. They were invited to complete the instrument before and after participating in four and after eight MCD sessions. The time between completing the first and the last questionnaire was for most respondents approximately 9 months. The Euro-MCD Instrument was distributed before MCD participation in 34 institutions, after 4 sessions in 32 and after 8 sessions in 23 institutions, as shown in the “Appendix: Characteristics Respondents”. The questionnaire was distributed on paper or by e-mail in Sweden and the Netherlands, and via a web-based questionnaire in Norway. A part of the responses to the instrument concerning perceived importance prior to MCD participation and more details on data collection have been published before (Svantesson et al. 2019; De Snoo-Trimp et al. 2017).

### Analysis of the Data

To present percentages for each answer option, ratings regarding perceived importance were descriptively analyzed using Statistical Package for Social Sciences (SPSS), version 22. To compare perceptions of importance *after* MCD participation with perceptions *before*, two statistical tests were used. Paired samples t-tests were used for individuals who completed both a questionnaire before as well as afterwards. Independent samples t-tests were performed to compare the (independent) group of respondents who completed only one questionnaire, either before or after MCD participation. As the ratings were not normally distributed, Wilcoxon signed-rank test and Chi-Square tests were used.

To examine the factor structure of the Euro-MCD Instrument, correlations between ratings for the 26 Euro-MCD items were analyzed using exploratory factor analyses. This statistical procedure explores a possible clustering of the data (the factor structure) by examining for which items there is a high correlations between answers. We looked for a factor structure that fits the data both before and after MCD participation.

Perceptions *after* participation in four and eight MCD sessions were merged in order to obtain sufficient power for comparing ratings before and after MCD participation and for the factor analyses. From respondents who rated the items both after 4 and 8 sessions (N = 129), their answers at the latest moment (i.e., after 8 sessions) were included in the analyses, because at that moment, they had gained more experience with MCD sessions, and based their assessment of items on a more extended and robust practice, thus covering also the sessions they had experienced when rating the items after 4 sessions.

### Ethical Considerations

Questionnaires were processed anonymously and participation was on a voluntary basis. At the start of the field study in Sweden, an advisory statement including “no objection to this study” was made by the Swedish Regional Ethical Review Board (dnr 2012/34). This statement was appropriate for Norway as well to perform the study, while the Norwegian Social Science Data Service was informed about the study. In the Netherlands, the Ethical Review Board was informed about the study and it was judged as not requiring further ethical review by law (2017.612).

## Results

The Euro-MCD Instrument was completed after participation in 4 MCD sessions by 443 healthcare professionals and after 8 sessions by 247 healthcare professionals. Before MCD participation, 756 professionals completed the instrument, of which 273 healthcare professionals completed it also after MCD participation (in 4 and/or 8 sessions). The characteristics of respondents including distributions over countries and healthcare domains are presented in the “Appendix: Characteristics Respondents”. In this section, the perceptions on important items after four and eight sessions will first be described, continued by a comparison with perceptions prior to participation and the results regarding the factor structure of the items.

### Perceptions on Important Items After MCD Participation

After participation in MCD, more than 69% of the healthcare professionals rated all items as ‘quite’ or ‘very’ important (see Table 1). On average, the answer option ‘Not important’ was chosen by only 3% per item (ranging from 0 to 5%) and the answer option ‘Somewhat important’ by 17% (ranging from 8 to 26%). The top-10 of items perceived as most important by most (82–91%) respondents included three items from the Euro-MCD domain Enhanced collaboration, namely ‘More open communication among co-workers’ (no. 2), ‘Better mutual understanding of each other’s reasoning and acting’ (no. 8) and ‘Enhances mutual respect amongst co-workers’ (no. 24). Two items concerned the domain Improved Moral Reflexivity: ‘Develops my skills to analyze ethical difficult situations’ (no. 1) and ‘I see the situation from different perspectives’ (no. 9). Another two items concerned the domain Improved Moral Attitude: ‘I listen more seriously to other’s opinions’ (no. 17) and ‘I understand better what it means to be a good professional’ (no. 26). The remaining three items from these ten came from three different domains: Concrete Results (‘Find more courses of action to manage the situation’, no. 16), Impact on the Organizational Level ( ‘I and my co-workers become aware of recurring situations’, no. 10) and Enhanced Emotional Support (‘Enhances possibility to share difficult emotions and thoughts’, no. 15).

### Comparing Ratings After Participation with Those Before MCD Participation

The ratings *after* participation are similar to those of respondents *before* participation, as also *before* participation the majority (more than 75%) of respondents rated all items as ‘quite’ or ‘very’ important and here even less respondents chose the option ‘Not important’ (average of 2%, ranging from 0 to 5%) or ‘Somewhat important’ (average of 13%, ranging from 4 to 21%). The top-10 of most important items prior to participation is similar to the top-10 after MCD participation as just described, except for the items from the domain Improved Moral Attitude (nos. 17 and 26). These items from the top-10 after MCD participation did *not* appear in the top-10 of most important items prior to participation. Instead, *before* MCD participation, two other items were highly rated: one from the domain Enhanced Collaboration (‘I and my co-workers manage disagreements more constructively’, no. 22) and another one from the domain of Concrete Results (‘Consensus is gained amongst co-workers in how to manage the situation’, no. 3).

Considering the difference in ratings of perceived importance before and after MCD participation, respondents perceived most (21 out of 26) items as *less* important after participation than before, of which 16 changed significantly (see Table 1). These 16 items included all items from the domains Concrete Results and Impact on the Organizational Level, and almost all items from the domains of Improved Moral Reflexivity and Enhanced Collaboration. Significant differences concerned a mean change of 7% in responses of ‘quite’ and ‘very’ important, ranging from 4% for the item ‘More open communication among co-workers’ to 10% for the item ‘Concrete actions to manage the situation’. However, the majority of respondents (ranging from 70 to 91%) still rated these 16 items as ‘quite’ or ‘very’ important after participation in MCDs. For instance, the item ‘More open communication among co-workers’ was perceived as ‘quite’ or ‘very’ important by 96% before and by 91% after participation in MCD sessions. Hence, the significant differences in the importance ratings were not considered meaningful.

On average, 43 respondents (10%) and 30 respondents (12%) did not give any answer or chose the option ‘Cannot take a stand’ after 4 or respectively 8 sessions. This number was 21 respondents (3%) before MCD participation. In Table 1, outcomes were marked where more than 10% of respondents did not complete the item or answered ‘Cannot take stand’. In particular, three items had relatively high percentages for ‘Cannot take stand’ or missings on all moments: ‘I listen more seriously to other’s opinions’ (no. 17, 7% before, 13% after 4 sessions and 13% after 8 sessions); ‘I and my co-workers manage disagreements more constructively’ (no. 22, 4% before, 13% after 4 and 13% after 8 sessions) and ‘Better understanding of being a good professional’ (no. 26, 4% before, 13% after 4 and 17% after 8 sessions).

### Factor Structure of Importance Ratings of Euro-MCD Instrument

Factor analyses did not reveal the original categorization into six Euro-MCD domains. By examining the correlations between ratings, the factor analyses suggested a clustering into three domains. This was the case in both the ratings *before* MCD participation as well as *after* participation.

Yet, some similarities can be detected between the original Euro-MCD categorization and the clusters as revealed by factor analysis: all items from the domain Improved Moral Reflexivity were associated with each other (i.e., found in the same cluster), this was also the case for most items of the domain Enhanced Collaboration. Items from the domain Improved Moral Attitude were associated with those from the domain Enhanced Emotional Support. Furthermore, the items from the domains of Concrete Results and Impact on Organizational Level did not clearly cluster together.

In the final clusters, 24 out of 26 items were found in a cluster *before* MCD participation and 25 out of 26 items were found in a cluster *after* MCD participation. Table 2 presents the correlations for each item and thereby shows the clusters of items for both factor analyses (before and after MCD participation).

The items ‘Consensus is gained amongst co-workers in how to manage the situation’ (no. 3) and ‘I gain more clarity about own responsibility in difficult situations’ (no. 23) did not associate with other items in the ratings *before* MCD participation. Here, the item ‘I listen more seriously to other’s opinions’ (no. 17) correlated with items of more than one cluster.

With regard to the factor analyses *after* MCD participation, the item ‘Enables me to better manage the stress from the ethical situation’ (no. 4) did not associate with any item at any cluster, and seven items were still associated with items from more than one cluster. Many items were distributed over the same clusters when compared with the classification of the responses *before* MCD participation.

In total, 16 out of 26 items were correlated with each other according to the same classification *before* and *after* MCD participation. The final factor models with clusters of items, with reference to their Euro-MCD domain, are shown in Table 3. In the first cluster, five items correlate with each other in the same way both *before* and *after* MCD participation. This factor seems to involve the individual feelings, emotions and attitude as these items come from the Euro-MCD domains Enhanced Emotional Support and Improved Moral Attitude, indicating that these domains are related to each other. In the second cluster, seven items are clustered similarly, which concern the awareness of and skills to identify, analyze and act upon ethically difficult situations. These items include all items from the domain of Moral Reflexivity and two from the domains Concrete Results and Impact on Organizational Level: ‘Find more courses of action to manage the situation’ and ‘I and my co-workers become more aware of recurring situations’. This confirms the link among items of Improved Moral Reflexivity. This cluster also indicates a need to reconsider the items in the domains Concrete Results and Impact on Organizational Level as they might not be interpreted according to the intended meaning. The third cluster seems to concern the teamwork among co-workers since it consists of four items, all from the Euro-MCD domain Enhanced Collaboration. For this domain, the presupposed associations between items are also confirmed.

**Table 3:** Overview outcomes per cluster compared to Euro-MCD domains

Cluster	No. item Euro-MCD Instrument	Euro-MCD domain^	
**1**	Clustered outcomes both *before* and *after* MCD participation	17. I listen more seriously to other’s opinions*	4
		18. Increases awareness of own emotions*	1
		19. Strengthens my self-confidence when managing difficult situations*	1
		25. I become more aware of my preconceived notions*	4
		26. I understand better what it means to be a good professional	4
	Only *before* MCD participation	4. Enables me to better manage the stress from the ethical situation	1
		6. Gives me more courage to express my ethical standpoint	4
		7. I feel more secure to express doubts or uncertainty regarding difficult situations	1
	Only *after* MCD participation	15. Enhances possibility to share difficult emotions and thoughts	1
		21. I and my co-workers examine more critically existing practice/policies in workplace	5
		22. I and my co-workers manage disagreements more constructively	2
		23. I gain more clarity about own responsibility in difficult situations*	4
		24. Enhances mutual respect amongst co-workers*	2
**2**	Clustered outcomes both *before* and *after* MCD participation	1. Develops my skills to analyze ethical difficult situations	3
		9. I see the situation from different perspectives*	3
		10. I and my co-workers become more aware of recurring situations*	5
		11. Increases my awareness of the complexity of the situation	3
		12. Enhances my understanding of ethical theories	3
		16. Find more courses of action to manage the situation	6
		20. Develops my ability to identify the core ethical question in difficult situations	3
	Only *before* MCD participation	5. Contributes to the development of practice/policies in the workplace	5
		13. Enables to decide on concrete actions to manage the situation	6
		21. I and my co-workers examine more critically existing practice/policies in workplace	5
	Only *after* MCD participation	18. Increases awareness of own emotions*	1
		19. Strengthens my self-confidence when managing difficult situations*	1
		23. I gain more clarity about own responsibility in difficult situations*	4
		25. I become more aware of my preconceived notions*	4
**3**	Clustered outcomes both *before* and *after* MCD participation	2. More open communication among co-workers	2
		8. Better mutual understanding of each other’s reasoning and acting	2
		14. Greater opportunity for everyone to have their say	2
		24. Enhances mutual respect amongst co-workers	2
	Only *before* MCD participation	15. Enhances possibility to share difficult emotions and thoughts	1
		17. I listen more seriously to other’s opinions*	4
		22. I and my co-workers manage disagreements more constructively	2
	Only *after* MCD participation	3. Consensus is gained amongst co-workers in how to manage the situation	6
		5. Contributes to the development of practice/policies in the workplace	5
		6. Gives me more courage to express my ethical standpoint	4
		7. I feel more secure to express doubts or uncertainty regarding difficult situations	1
		9. I see the situation from different perspectives*	3
		10. I and my co-workers become more aware of recurring situations*	5
		13. Enables to decide on concrete actions to manage the situation	6
**Not associated with any cluster** *before* MCD		3. Consensus is gained amongst co-workers in how to manage the situation	6
		23. I gain more clarity about own responsibility in difficult situations	4
*After* MCD		4. Enables me to better manage the stress from the ethical situation	1

*Correlated at > 1 cluster

**^Original Euro-MCD domains:**

1 = Enhanced emotional support

2 = Enhanced collaboration

3 = Improved moral reflexivity

4 = Improved moral attitude

5 = Impact on organizational level

6 = Concrete results

## Discussion

This paper described the importance of MCD outcomes according to healthcare professionals *after* MCD participation, a comparison with the perceived importance *before* MCD participation and results from the factor analyses on all rated outcomes in order to further develop the Euro-MCD Instrument.

### Perceptions on Importance: Reconsidering the Question in the Euro-MCD Instrument

Our study firstly showed that the majority of healthcare professionals, who completed the Euro-MCD Instrument, perceived all outcomes as quite or very important with only a very few respondents rating outcomes as not important. Their perceptions for 16 out of 26 outcomes did change significantly after participation in a series of MCD yet these changes were not clinically relevant. Outcomes perceived as most important mainly concerned the domain of Enhanced Collaboration, including open communication, mutual understanding and respect, and outcomes referring to the domain Improved Moral Reflexivity, like being able to see the situation from various perspectives. These outcomes are in line with literature on underlying hermeneutical fundamentals of MCD and goals of CES in general (Metselaar et al. 2015; Porz et al. 2011; Widdershoven and Molewijk 2010): “Clinical ethics […] does support individual professionals in becoming more sensitive to moral issues and groups of professionals in dealing with difficult situations by improving communication and dialogical learning” (Widdershoven and Molewijk 2010, p. 51). Furthermore, our findings are in line with previous evaluation studies (Haan et al. 2018; Janssens et al. 2014; Hem et al. 2014; Silén et al. 2014; Weidema et al. 2013). Based on 25 empirical studies on impact of MCD, Haan et al. (2018) concluded that most changes concerned the interaction and understanding of perspectives among healthcare professionals (i.e., collaboration) and the “awareness of the moral dimension of one’s work and awareness of the importance of reflection” (i.e., moral reflexivity).

Our study adds to existing literature on importance of MCD outcomes that also *after* participation in MCD, most respondents perceive outcomes as of quite or high important. The finding that all outcomes were perceived as important by the majority of respondents *before* participating in MCD has also been described in previous Euro-MCD field studies (Svantesson et al. 2019; De Snoo-Trimp et al. 2017). A possible reason for these high rates both before and after MCD participation is that participants might have interpreted the question “How important is the outcome to you?” in (at least) two ways: “Do you feel the need for this outcome?” or “Do you expect that MCD would lead to this outcome?”. This might explain the high rates prior to participation, where respondents just had high needs for certain MCD related outcomes or high expectations of what MCD could lead to. After participation in MCD, respondents might perceive outcomes also as highly important to (still) stress the need for MCD related outcomes or to express that MCD indeed leads to these outcomes according to their expectations.

Our findings further showed that 16 outcomes were perceived as significantly less important *after* participation in MCD than *before*. A reason for this might be that respondents considered some outcomes as less relevant when learning what MCD really is, as they had no idea prior to participation, or because they had too high expectations beforehand and adjusted these afterwards. Although these changes are statistically significant, they are small and we do not consider them as meaningful and clinically relevant changes. Note that these outcomes were still perceived as quite or very important by the majority (> 70%) of respondents. For instance, almost all outcomes from the domains of Moral Reflexivity and Enhanced Collaboration changed significantly but were still rated as the most important after MCD participation.

With regards to further development of the Euro-MCD Instrument, our findings indicate that the respondents—the healthcare professionals who take part in the MCD sessions—confirmed the importance and relevance of outcomes in the instrument and that they did not decisively differ in perceptions when asked for it (before or after MCD participation). Since respondents did not obviously discriminate among the presented outcomes, it would not be possible to tailor the content of MCD to prioritized outcomes or to weigh experienced outcomes against the prioritized outcomes. Hence, the usefullness of the question on perceived importance is not so clear anymore. We can, therefore, conclude that the question on perceived importance needs reconsideration and perhaps might even not be necessary in the future revision of the Euro-MCD instrument.

### Testing the Factor Structure of Euro-MCD Items on Perceived Importance

Secondly, our study showed that the presupposed categorization of outcomes into six domains was not confirmed in the factor analyses, but that three distinct clusters with 16 outcomes can be recognized. Yet, the Euro-MCD domains Improved Moral Reflexivity and Enhanced Collaboration could be recognized in these factor analyses because most of their items were indeed associated with each other. These domains, therefore, seem to reflect separate constructs, either referring to individual moral skills (i.e., outcomes from Improved Moral Reflexivity) or group collaboration (i.e., outcomes from Enhanced Collaboration). Furthermore, the domains Improved Moral Attitude and Enhanced Emotional Support seemed to refer to the same underlying construct as their outcomes associated with each other in the same category. This correlation between outcomes of these two domains was also found in our previous study concerning the factor structure of items regarding *experienced* MCD outcomes, both during the MCD sessions and beyond the MCD sessions in daily practice (De Snoo-Trimp et al. 2019). Considering this, we think that outcomes in these two Euro-MCD domains refer to individual virtues in which feelings and character aspects play a role, as we also suggested in our previous study (De Snoo-Trimp et al. 2019). Lastly, the domains of Concrete Results and Impact on Organizational Level were not clearly reflected in the factor models, indicating a need to reconsider and revise these domains. In these domains, some outcomes might have been unclear by having different meanings, resulting in a lack of correlations with other outcomes. For instance, the outcome ‘Consensus is gained amongst co-workers in how to manage the situation’ was associated with items of two clusters. It might have been interpreted as a collaboration-outcome by respondents: “*we as a group* reached consensus”, while it originally refers to Concrete Results and was intended to assess the joint *ability to concretely manage* the situation. It might, however, be a question if consensus should be an outcome of MCD at all as it is not as such emphasized in the literature on fundamentals and goals of MCD (Metselaar et al. 2015; Widdershoven and Molewijk 2010). Normative decisions (i.e., on what *should* be an outcome and why) need to be made in the further revision of these outcomes.

To conclude this part, the factor analyses from both this study and our previous study on experienced MCD outcomes (De Snoo-Trimp et al. 2019) provided important insights in the associations of the Euro-MCD domains, to be used in the future revision of the instrument. Our finding that 16 outcomes showed similar correlations in both studies indicates that these outcomes are representing the same construct (i.e., domain). For example, if respondents find teamwork important, they scored also high on the items ‘More open communication among co-workers’ and ‘Enhanced mutual respect amongst co-workers’. The finding of the same clustering in studies about perceived importance and experienced outcomes means that the questions are interpreted similarly. This also holds for different moments (i.e., before and after participation) and different settings (i.e., during the MCD sessions and after the MCD sessions in daily practice).

### Strengths and Limitations

A strength of this study is that we performed the current and other field studies with an open mind, not being reluctant to criticize the original structure and outcomes, which is important when developing or revising measurement instruments (De Vet et al. 2011). Another strength of this study is the large and heterogeneous population in which we could test the Euro-MCD Instrument, as the instrument intends to be applicable in various settings and contexts in which MCD is done (Svantesson et al. 2014). A limitation, however, is that because of this heterogeneity (in countries, settings and professional backgrounds), the number of respondents per subgroup was too small to allow for subgroup comparisons (e.g., the Dutch versus the Swedish or Norwegian respondents). We did not consider this a major weakness, as comparisons of subgroups was not the aim of this study. Another limitation is the limited data on perceived importance after participation in eight MCD sessions. Therefore, we had to merge the answers after four and eight sessions to obtain sufficient power for the factor analyses. As a consequence, this study does not show if respondents change their perceptions of importance when their participation in MCD develops further (i.e., between four and eight MCD sessions).

### Relevance

This study contributes to the empirical evidence (Svantesson et al. 2019; De Snoo-Trimp et al. 2019, 2017) for revising the Euro-MCD Instrument as a profound tool for measuring outcomes of MCD. Insight in participants’ perceptions of importance is crucial in this process since, in the end, they are the ones who should benefit from MCD. MCD, like any CES service, aims to improve quality of care mainly by supporting healthcare professionals in dealing with ethically difficult situations. Input from participants themselves is, therefore, important to define suitable outcomes that they are able to recognize, value and experience.

Insight into the factor structure of responses is highly relevant for further development of the instrument. Validated dimensions of outcomes (i.e., categories) will facilitate future use of the instrument as results can be presented per domain instead of per outcome, and these results will also become more reliable if a domain is measured by multiple related outcomes. As already stated by the developers, it is important “to know *if* there is a systematic pattern of MCD outcomes within the Euro-MCD” (Svantesson et al. 2014). Furthermore, since the Euro-MCD instrument consists of a rather long list of 26 outcomes, one of the aims of the field study was to reduce the number of outcomes to make it a feasible and easy-to-use tool for practice (Svantesson et al. 2014). The current findings, therefore, form valuable information for reducing outcome as it showed, for instance, that some outcomes showed hardly any correlation with any of the other outcomes and thus need thorough reconsideration.

## Conclusion

This study confirmed that also after MCD participation, healthcare professionals gave high rates to the importance of Euro-MCD outcomes. Findings indicate the need to reconsider whether we should still include the question on perceived importance in the revised Euro-MCD Instrument as well as the initial categorization of outcomes into six domains. Thus, the study contributes to the empirical evidence for the revision of the instrument. In this revision process, empirical evidence will be combined with researchers’ reflections, dialogues and theoretical justifications. This integration of empirical evidence and theoretical reflections will ultimately determine what outcomes *should* be MCD outcomes and why, and how these should be included in the instrument. The revised Euro-MCD Instrument will be published elsewhere in the near future.

## Data Availability

The datasets generated during and/or analysed during the current study are available from the corresponding author on reasonable request.

## Appendix: Characteristics Respondents

See Table

**Table 4:** Characteristics respondents Euro-MCD Instrument

	Before MCD	After 4 MCDs	After 8 MCDs
Total N	756	443	247
Country N (%)			
Sweden	275 (36)	130 (29)	142 (58)
NL	384 (51)	232 (52)	53 (21)
Norway	97 (13)	82 (18)	52 (21)
Male/female (%)	24/76	20/80	13/87
Age, mean (range)	44 (20–68)	45 (21–75)	45 (20–65)
Years of experience, mean (range)	18 (0–50)	18 (0–45)	19 (1–45)
Profession N (%)			
Nurse^a^	342 (45)	160 (37)	126 (53)
Nurse assistant	120 (16)	73 (17)	58 (24)
Doctor/specialist/psychiatrist	49 (7)	18 (4)	6 (3)
Therapist^b^	143 (19)	121 (28)	23 (9)
Manager^c^	45 (6)	32 (7)	19 (8)
Others^d^	47 (6)	28 (7)	6 (3)
Respondents per setting N (%)			
Community care services	137 (18)	110 (25)	77 (31)
Somatic hospital care	342 (45)	140 (32)	119 (48)
Psychiatric care	213 (28)	148 (33)	31 (13)
Mentally disabled care	49(7)	26 (6)	12 (5)
Health inspection/research	15 (2)	19 (4)	8 (3)
Number of involved institutions	34	30	25
MCD participation, mean (range)	0 (0–5)	3 (0–6)	4 (0–10)
Missing MCD participation (%)	30	60	51

^a^Including registered nurses; support workers and psychosocial workers

^b^Including physiotherapists; psychologists; spiritual caregivers; social workers

^c^Including head of departments and policy makers

^d^Including volunteers, clients, researchers, trustees, secretary and interns

4.
